# Supplementary material for: Monovalent metal ion binding promotes the first transesterification reaction in the spliceosome
Source: Nat Commun. 2023 Dec 20;14:8482. doi: 10.1038/s41467-023-44174-2 (PMC10733407; doi:10.1038/s41467-023-44174-2)
Supplement: Supplementary file 1 — Supplementary Information [file 41467_2023_44174_MOESM1_ESM.pdf]

Supplementary Information for

Monovalent metal ion binding promotes the first transesterification reaction in the spliceosome

Jana Aupič[1], Jure Borišek[2], Sebastian M. Fica[3], Wojciech P. Galej[4], Alessandra Magistrato[1,*]

[1] National Research Council of Italy (CNR) - Materials Foundry (IOM) c/o International School for Advanced Studies (SISSA), Trieste, Italy

[2] Theory department, National Institute of Chemistry, Ljubljana, Slovenia

[3] Department of Biochemistry, University of Oxford, Oxford, UK

[4] European Molecular Biology Laboratory, Grenoble, France

*Corresponding author, e-mail: alessandra.magistrato@sissa.it

This PDF file contains:

Supplementary Discussion

Supplementary Figs. 1-19

Supplementary Table 1

Supplementary Discussion

The blue moon ensemble approach to computing the free energy profile

In the blue moon ensemble formalism the Helmholtz free energy difference (ΔF) is obtained by integrating over the derivative of the potential of the mean force W with respect to the reaction coordinate ξ which is constrained at different values in each simulation window.

$$\Delta F = \int^{\xi} \frac{dW}{d\xi} d\xi \tag{1}$$

The derivative of mean force in Eq. 1 can be expressed as

$$\frac{dW}{d\xi} = \frac{\langle Z^{-1/2}[-\lambda + k_{\mathrm{B}}TG]\rangle_{\xi}}{\langle Z^{-1/2}\rangle_{\xi}}, \tag{2}$$

where λ is the Lagrangian multiplier that corresponds to the strength of the constrained force, k_{B} is the Boltzmann constant, T is the temperature and Z and G are configurational factors that compensate for the bias introduced by the constraint.

$$Z = \sum_{i} \frac{1}{m_i} \left(\frac{\partial \xi}{\partial \mathbf{r}_i}\right)^2 \tag{3}$$

$$G = \frac{1}{Z^2} \sum_{i,j} \frac{1}{m_i m_j} \frac{\partial \xi}{\partial \mathbf{r}_i} \cdot \frac{\partial^2 \xi}{\partial \mathbf{r}_i \partial \mathbf{r}_j} \cdot \frac{\partial \xi}{\partial \mathbf{r}_j} \tag{4}$$

Above, m_i and $\mathbf{r}_i$ stand for mass and coordinates of atom i. Indices i and j run over all of the particles in the system.

For certain simple reaction coordinates, Z is a constant, G equals zero and Eq. 2 simplifies to

$$\frac{dW}{d\xi} = -\langle \lambda \rangle_{\xi} \,. \tag{5}$$

However, when the reaction coordinate is the difference of two interatomic distances that share a common atom,

$$\xi = |\mathbf{r}_i - \mathbf{r}_j| - |\mathbf{r}_j - \mathbf{r}_k|, \tag{6}$$

as in our case, G equals zero, while

$$Z = \frac{1}{m_i} + \frac{1}{m_k} + \frac{2}{m_j}(1 - \cos \alpha), \tag{7}$$

where α is the angle between the atoms i, j and k.

In our simulations, the fluctuations in the value of the angle α were minimal and as a result Z was effectively a constant, making the contribution of the correction term negligible (< 1 %).

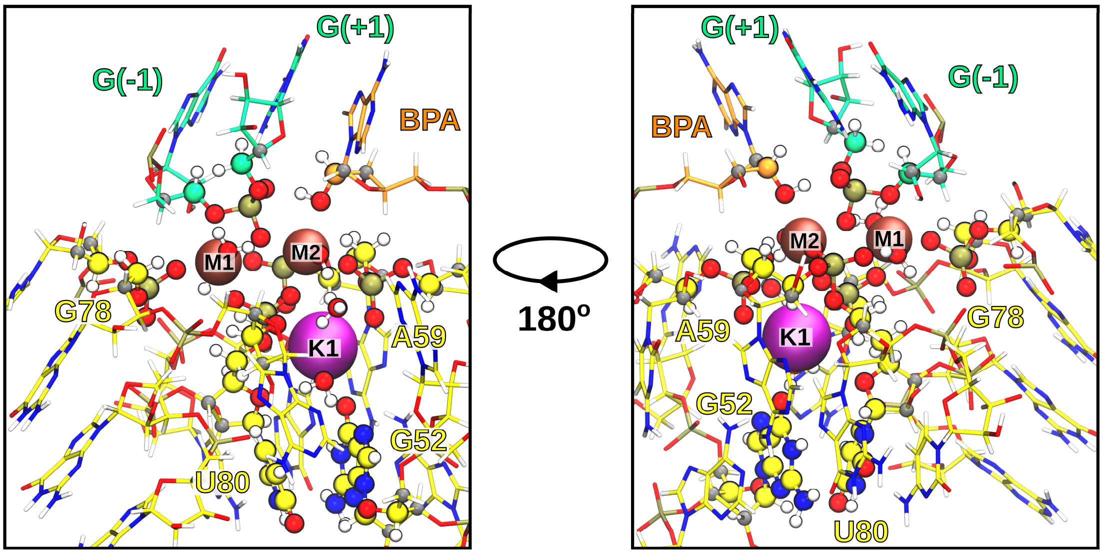

Supplementary Figure 1. Partitioning of the system between the quantum (QM) and the classical (MM) part during QM/MM molecular dynamics (MD) simulations. Atoms in the QM zone are shown as van der Waals spheres, while grey spheres denote link atoms. The MM region is displayed as lines.

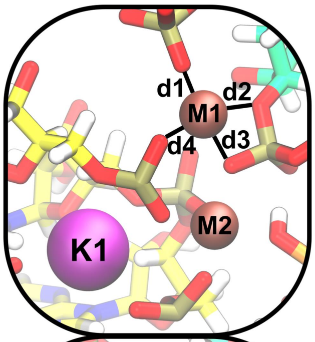

	+Yju2, +Cwc25		+Yju2, -Cwc25		-Yju2, -Cwc25	
	avr (nm)	std (nm)	avr (nm)	std (nm)	avr (nm)	std (nm)
d1	0.20	0.01	0.20	0.01	0.19	0.01
d2	0.23	0.01	0.23	0.01	0.22	0.01
d3	0.23	0.01	0.24	0.02	0.23	0.01
d4	0.20	0.01	0.20	0.01	0.20	0.01

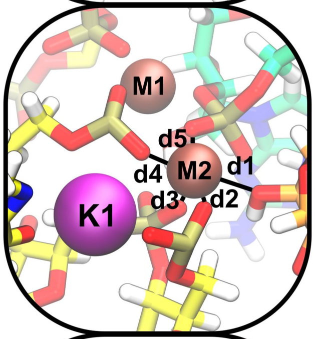

	+Yju2, +Cwc25		+Yju2, -Cwc25		-Yju2, -Cwc25	
	avr (nm)	std (nm)	avr (nm)	std (nm)	avr (nm)	std (nm)
d1	0.23	0.01	0.25	0.02	0.32	0.07
d2	0.21	0.01	0.21	0.01	0.21	0.01
d3	0.20	0.01	0.20	0.01	0.21	0.01
d4	0.21	0.01	0.21	0.01	0.20	0.01
d5	0.22	0.01	0.21	0.01	0.21	0.01

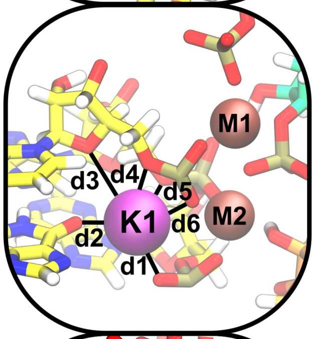

	+Yju2, +Cwc25		+Yju2, -Cwc25		-Yju2, -Cwc25	
	avr (nm)	std (nm)	avr (nm)	std (nm)	avr (nm)	std (nm)
d1	0.28	0.02	0.29	0.02	0.28	0.02
d2	0.28	0.02	0.28	0.02	0.28	0.01
d3	0.34	0.02	0.34	0.02	0.36	0.03
d4	0.29	0.01	0.29	0.02	0.28	0.02
d5	0.31	0.02	0.33	0.02	0.33	0.03
d6	0.29	0.02	0.30	0.01	0.29	0.02

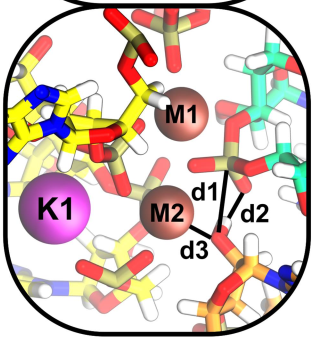

	+Yju2, +Cwc25		+Yju2, -Cwc25		-Yju2, -Cwc25	
	avr (nm)	std (nm)	avr (nm)	std (nm)	avr (nm)	std (nm)
d1	0.33	0.02	0.31	0.01	0.36	0.03
d2	0.30	0.02	0.21	0.05	0.24	0.06
d3	0.23	0.01	0.25	0.02	0.32	0.07

Supplementary Figure 2. Effect of splicing factors Yju2 and Cwc25 on the stability of the coordination sphere of Mg^{2+} ions (M1 and M2), the potassium ion (K1), and positioning of the nucleophilic hydroxyl group of the branch point adenosine (BPA) that carries out the nucleophilic attack on the scissile phosphate group (last panel). The average distances (avr) and corresponding standard deviations (std) were calculated over 5 ps of QM/MM MD simulations (n = 1000 frames). Source data are provided as a Source Data file.

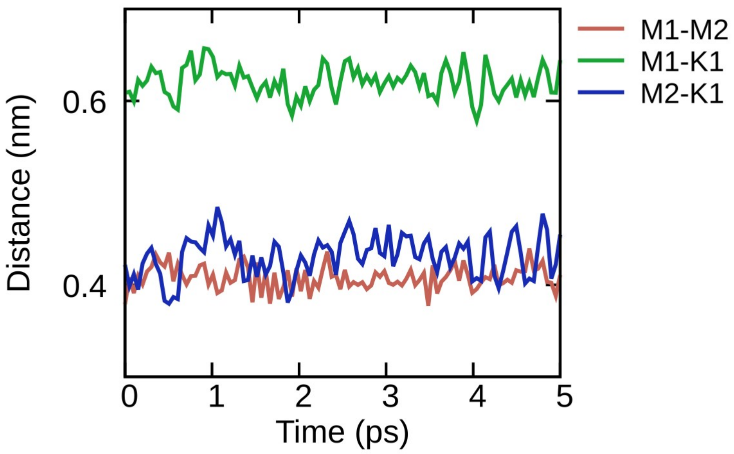

Supplementary Figure 3. Time evolution of distances between metal ions in the active site during unbiased QM/MM MD simulations of the spliceosome system containing both Yju2 and Cwc25 splicing factors. The average distance between Mg^{2+} ions (M1 and M2, brown line) was 4.1 ± 0.1 Å, while K^{+} (K1) was on average 4.3 ± 0.2 Å away from the closer Mg^{2+} ion (M2, blue line). Source data are provided as a Source Data file.

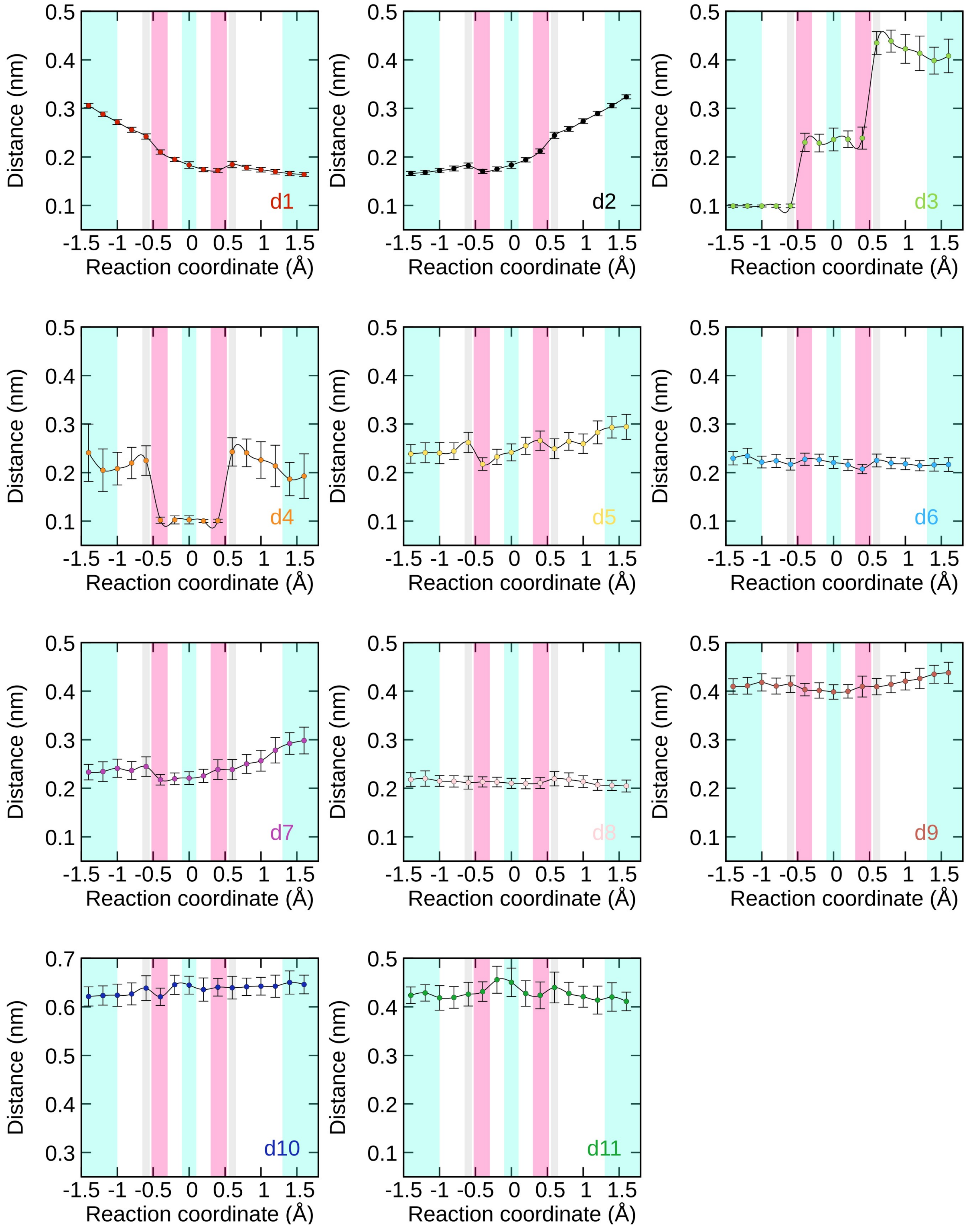

Supplementary Figure 4. Monitored distances between active site atoms during the branching reaction. The distances are as marked in Fig. 2a. Data are presented as mean values +/- SD (n = 1000 frames). Source data are provided as a Source Data file.

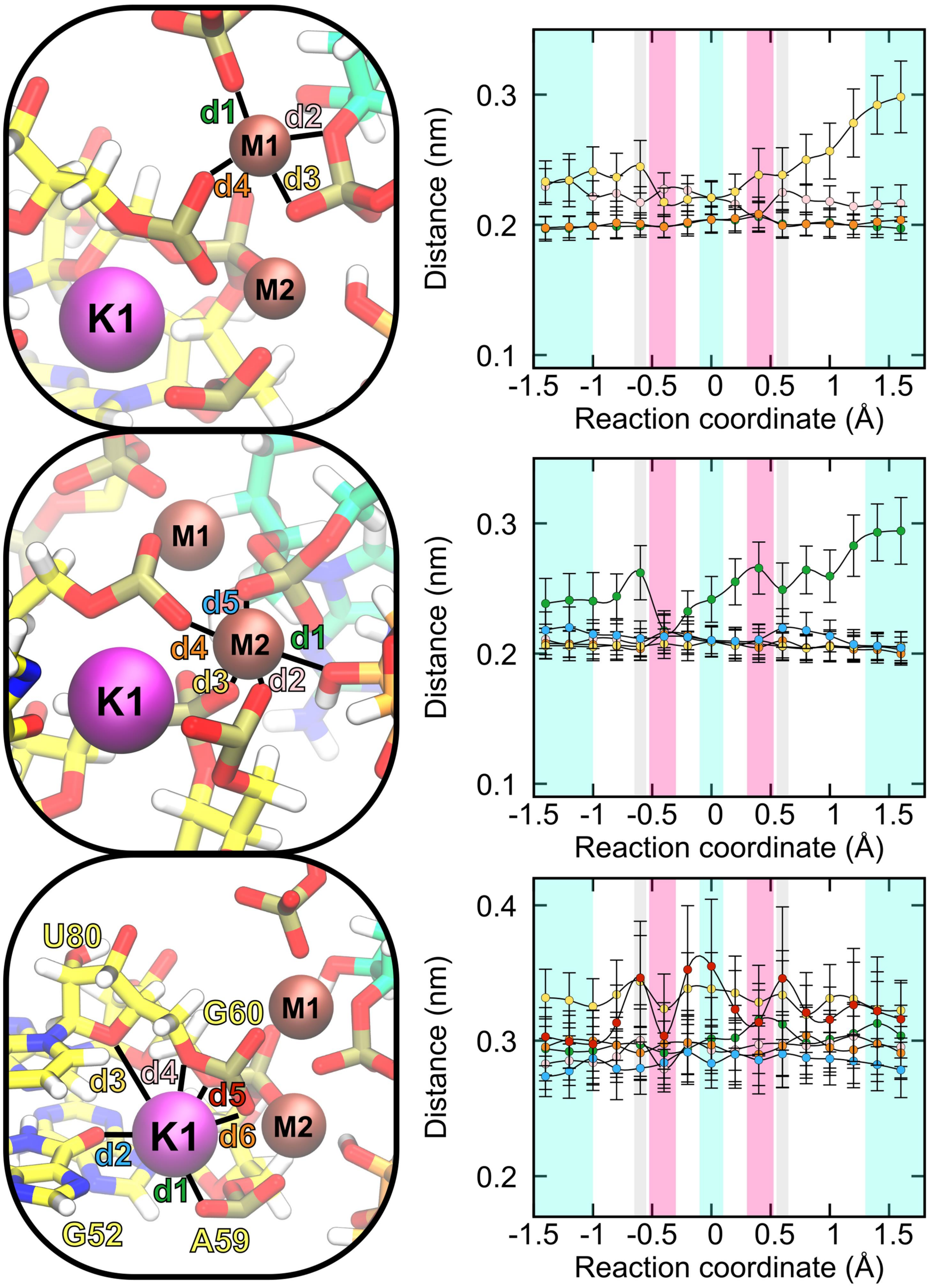

Supplementary Figure 5. Fluctuation of the coordination sphere of Mg^{2+} ions (M1 and M2) and the K^{+} ion (K1) as a function of the reaction coordinate employed during biased QM/MM MD simulations. The reaction coordinate was defined as the difference of the distance between O3' of the leaving group and phosphorous atom in the scissile phosphate group and the distance between the same phosphorous atom and the O2' of the BPA. Data are presented as mean values +/- SD (n = 1000 frames). Source data are provided as a Source Data file.

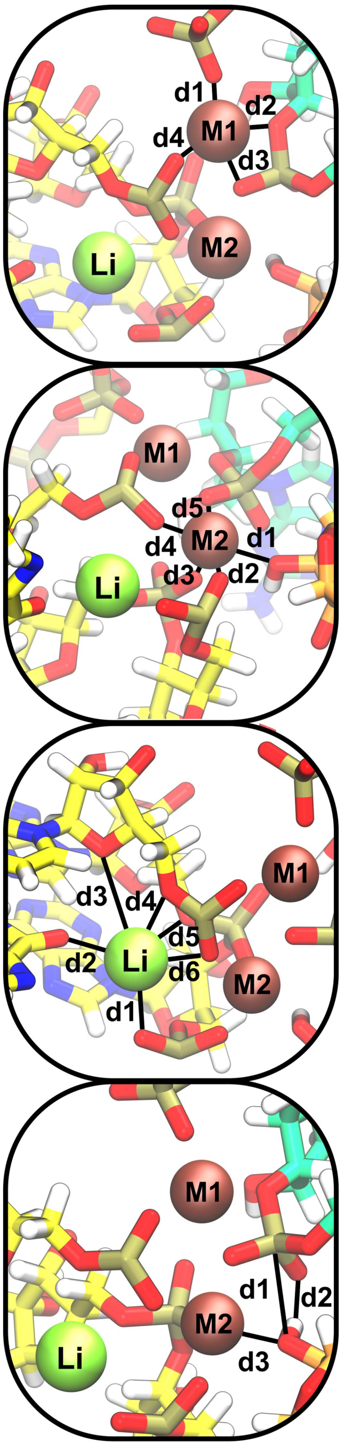

	avr (nm)	std (nm)
d1	0.20	0.01
d2	0.24	0.02
d3	0.23	0.02
d4	0.20	0.01

	avr (nm)	std (nm)
d1	0.29	0.05
d2	0.20	0.01
d3	0.21	0.01
d4	0.21	0.01
d5	0.22	0.01

	avr (nm)	std (nm)
d1	0.41	0.03
d2	0.21	0.03
d3	0.26	0.04
d4	0.25	0.03
d5	0.43	0.03
d6	0.37	0.03

	avr (nm)	std (nm)
d1	0.34	0.02
d2	0.20	0.04
d3	0.29	0.05

Supplementary Figure 6. Stability of the coordination sphere of Mg^{2+} ions (M1 and M2), the Li^{+} ion (Li), and positioning of the nucleophilic hydroxyl group of the BPA during unbiased QM/MM MD. The average distances (avr) and corresponding standard deviations (std) were calculated over 5 ps of simulation (n = 1000 frames). Source data are provided as a Source Data file.

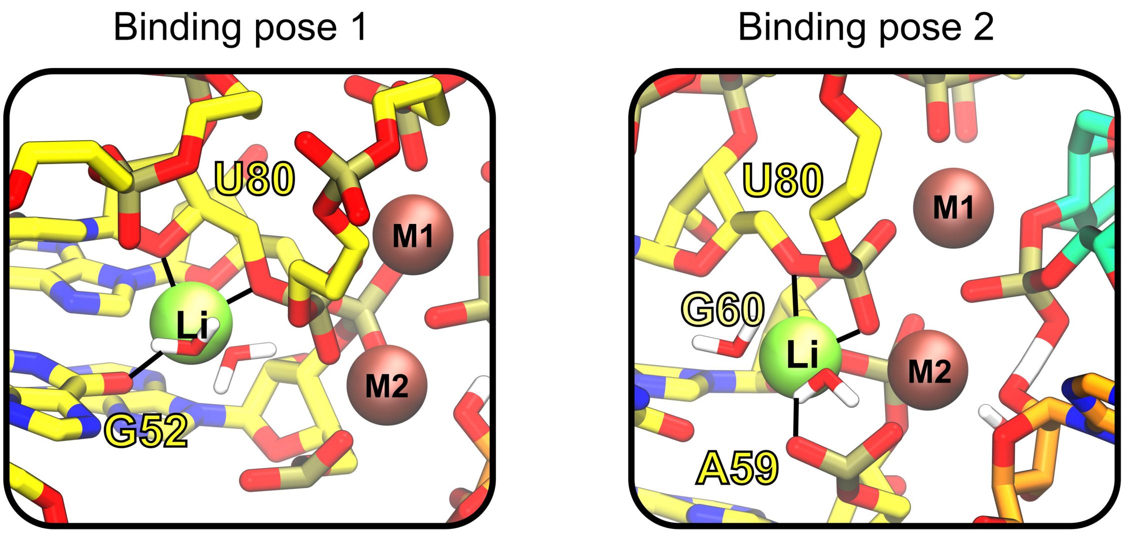

Supplementary Figure 7. Li^+ cannot stably coordinate at the K1 site, due to different ion size and coordination preferences. Li^+ ion fluctuates between two binding poses. In the first (left panel) Li^+ is coordinated by G52 and U80, while in the second pose (right panel), A59, G60 and U80 act as coordinating ligands.

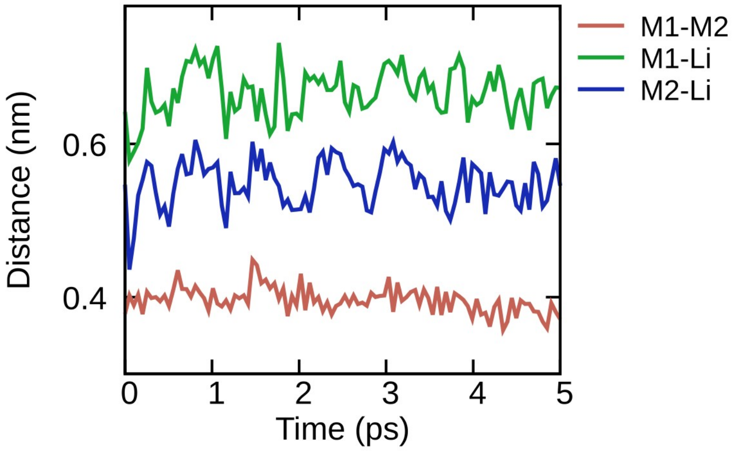

Supplementary Figure 8. Time evolution of distances between metal ions in the active site during unbiased QM/MM MD simulations with Li^{+} at the K1 site. The average distance between Mg^{2+} ions (M1 and M2, brown line) was 4.0 ± 0.2 Å, while Li^{+} was on average 5.5 ± 0.3 Å away from the closer Mg^{2+} ion (M2, blue line), significantly further compared to the K^{+} ion. Source data are provided as a Source Data file.

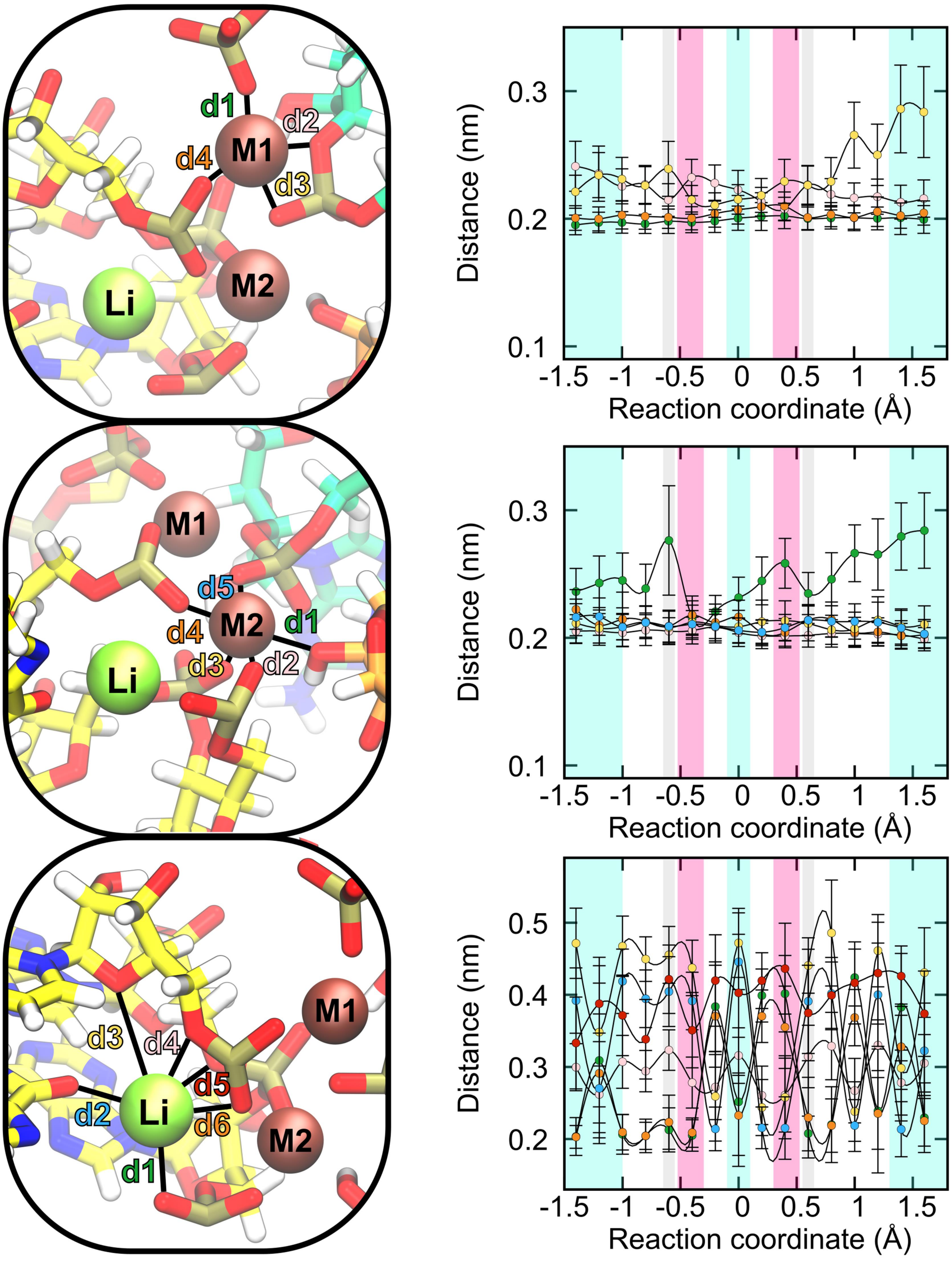

Supplementary Figure 9. Fluctuation of the coordination sphere of Mg^{2+} ions (M1 and M2) and the Li^{+} ion (Li) as a function of the reaction coordinate employed during biased QM/MM MD simulations. Data are presented as mean values +/- SD (n = 1000 frames). Source data are provided as a Source Data file.

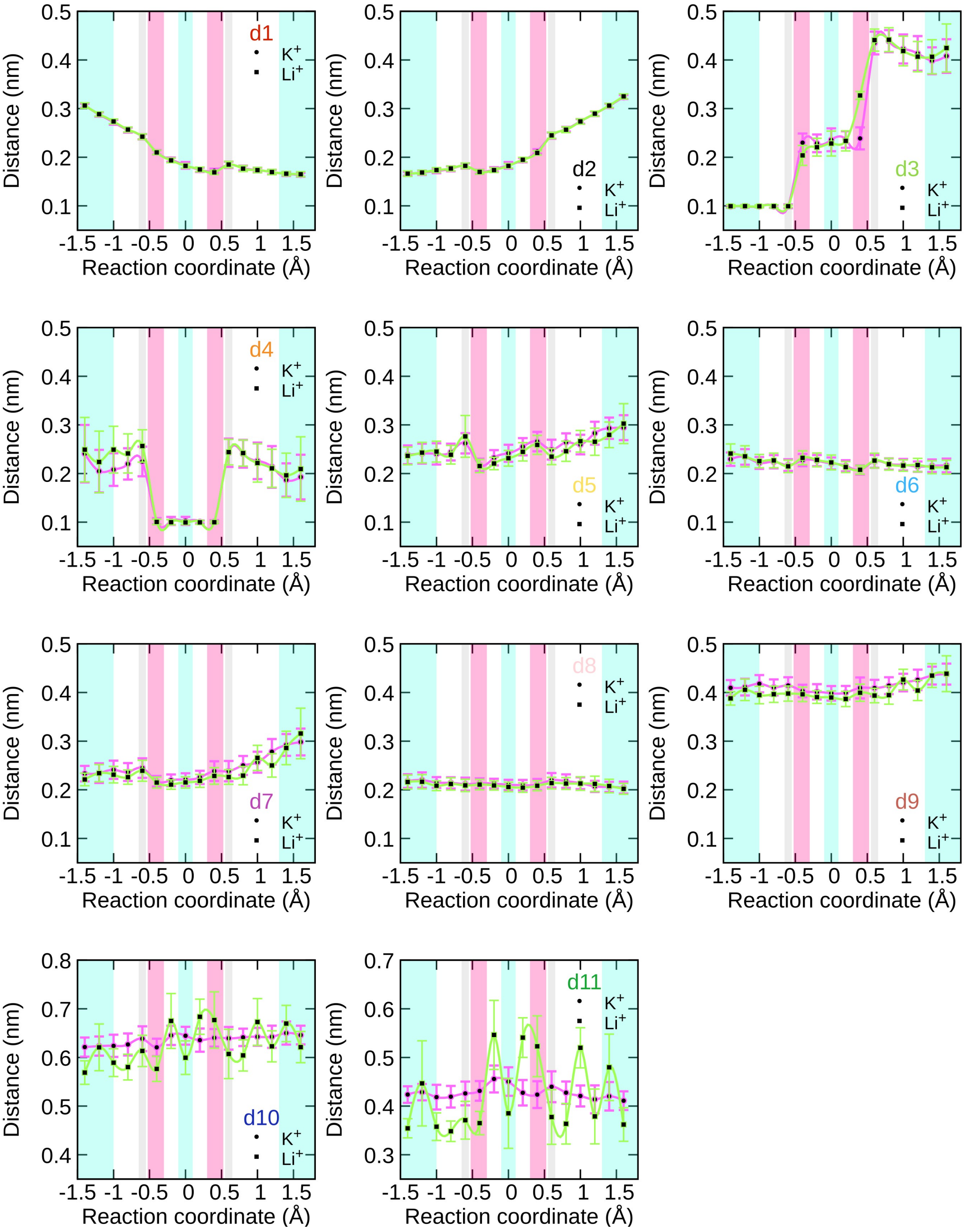

Supplementary Figure 10. Comparison of monitored distances during the branching reaction performed in the presence of K^+ (violet line) or Li^+ ion (green line). The distances are marked in Fig. 2a. Data are presented as mean values +/- SD (n = 1000 frames). Source data are provided as a Source Data file.

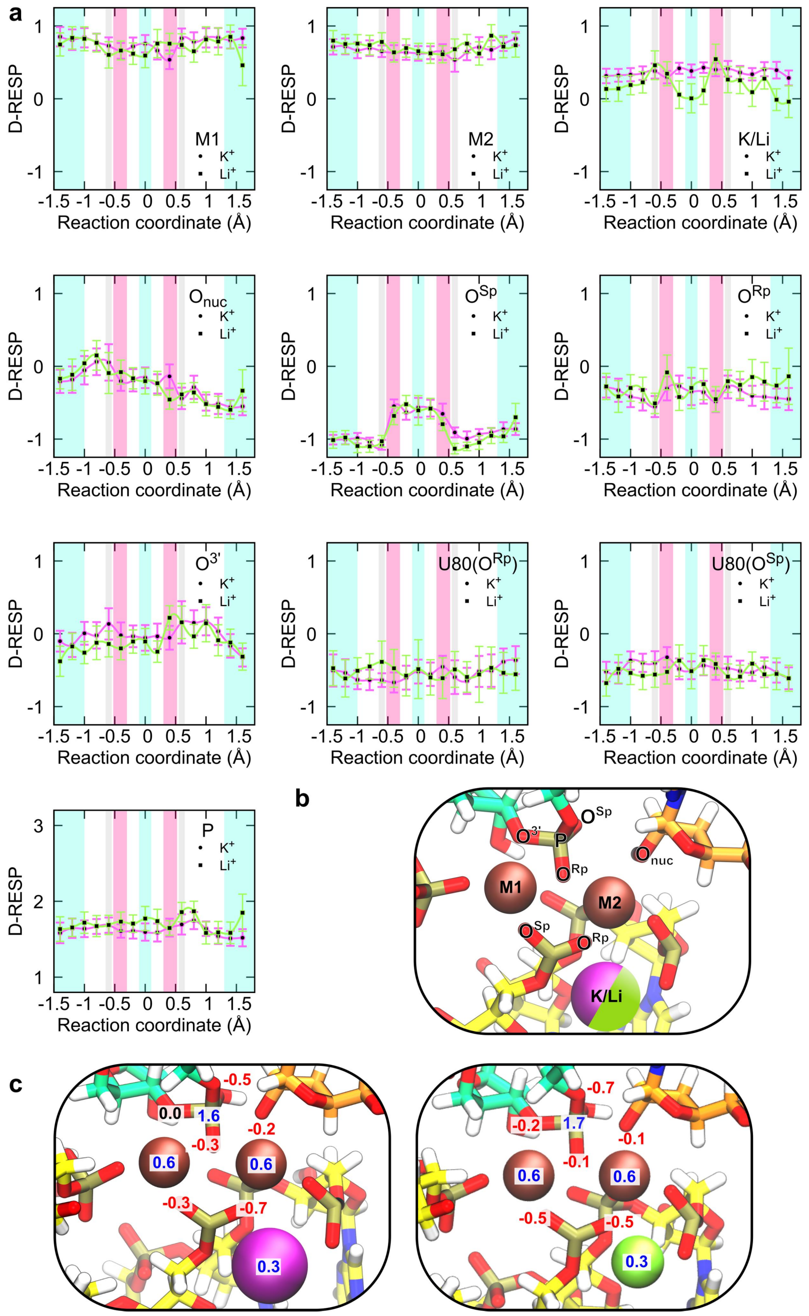

Supplementary Figure 11. a, D-RESP charges of atoms in the active site as a function of the reaction coordinate (RC). Charges were calculated both for simulations performed with the K^+ (violet line) and the Li^+ ion (green line). Data are presented as mean values +/- SD (n = 100 frames). **b,** Close up of the active site with atom labels. **c**, Comparison of partial charges on active site atoms in the rate-limiting transition state (RC = -0.4). In the presence of the K^+ ion, the distribution of charges is more favourable for the splicing reaction to occur (i.e. the O_{nuc} is more negatively charged and the leaving $O^{3'}$ is more neutral), however, the observed differences are within the margin of error. Source data are provided as a Source Data file.

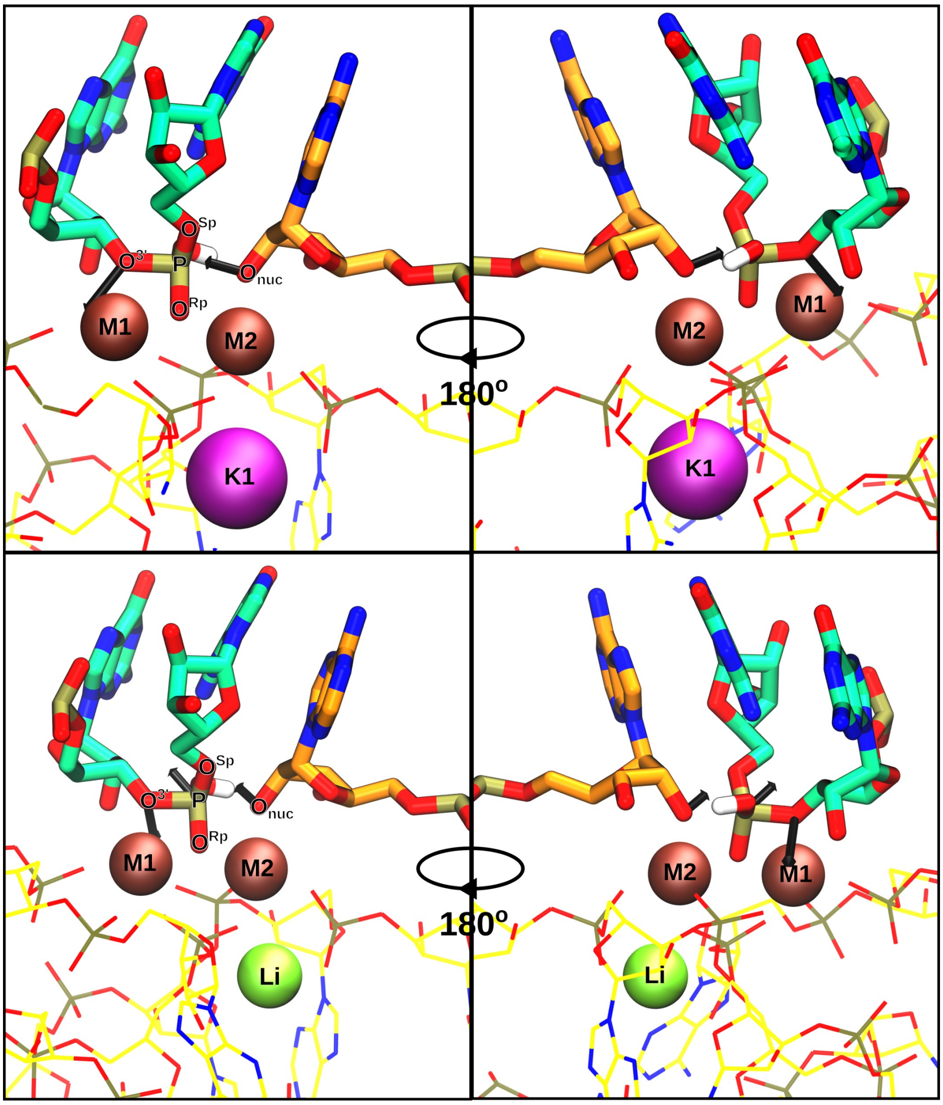

Supplementary Figure 12. Electric field forces acting on reactive atoms in the rate-limiting transition state (TS1). Top panels show the forces in the presence of the K^+ ion at the K1 site, while the bottom panels depict the forces obtained when K^+ was replaced by Li^+. In the presence of K^+, the force on G(+1)-P is too small to be noticeable.

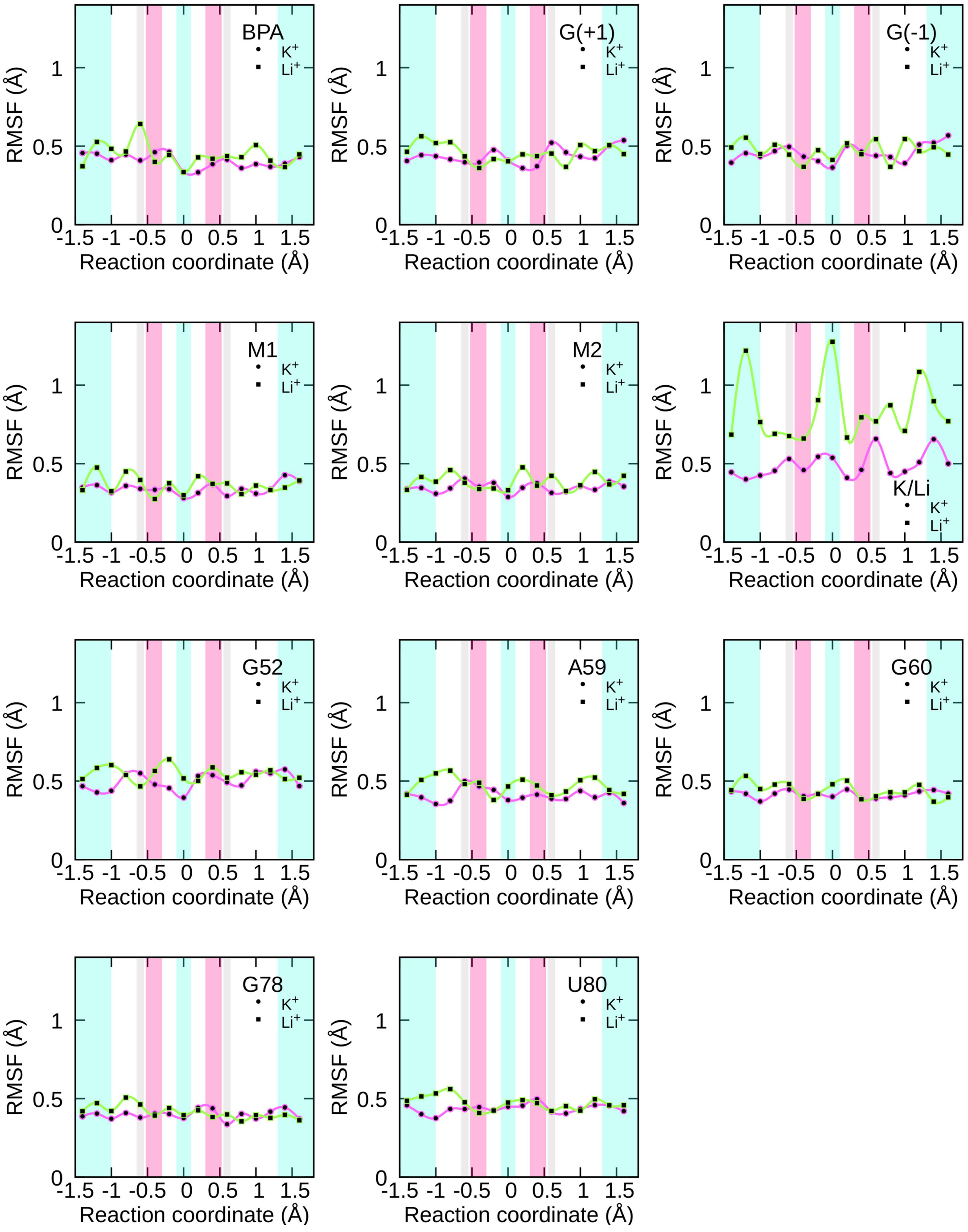

Supplementary Figure 13. Relative mean square fluctuation (RMSF) of residues involved in the splicing reaction as a function of the reaction coordinate. The violet line corresponds to simulations performed with the K^+ ion, while the green line reports results obtained when K^+ was replaced by the Li^+ ion. Data are calculated from 200 frames.

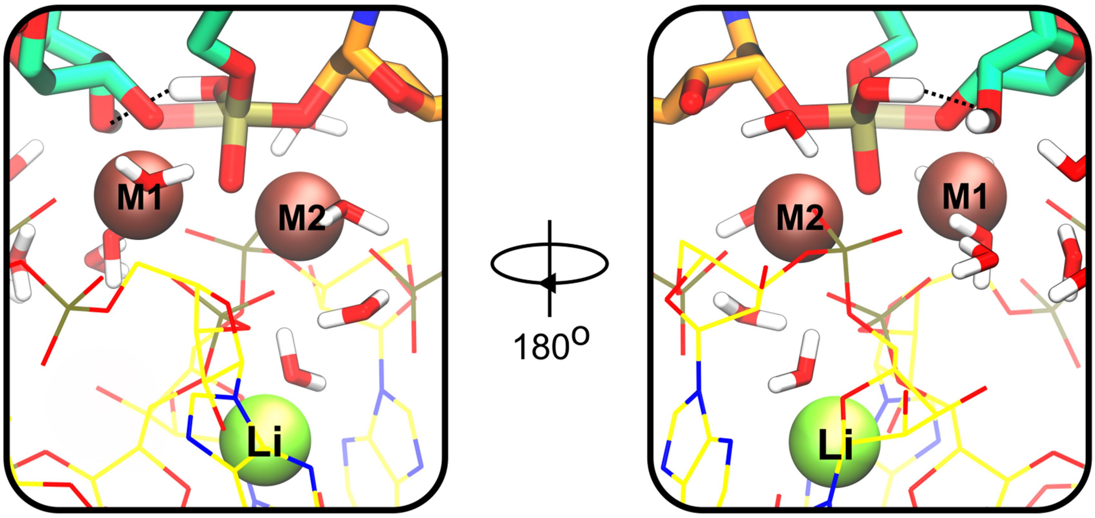

Supplementary Figure 14. Snapshot of the transition state TS2 in presence of the Li^+ ion in the active site. Hydroxyl group of the scissile phosphate is forming a hydrogen bond with the O2' of the G(-1) and not with a water molecule as observed in simulations performed with the K^+ ion.

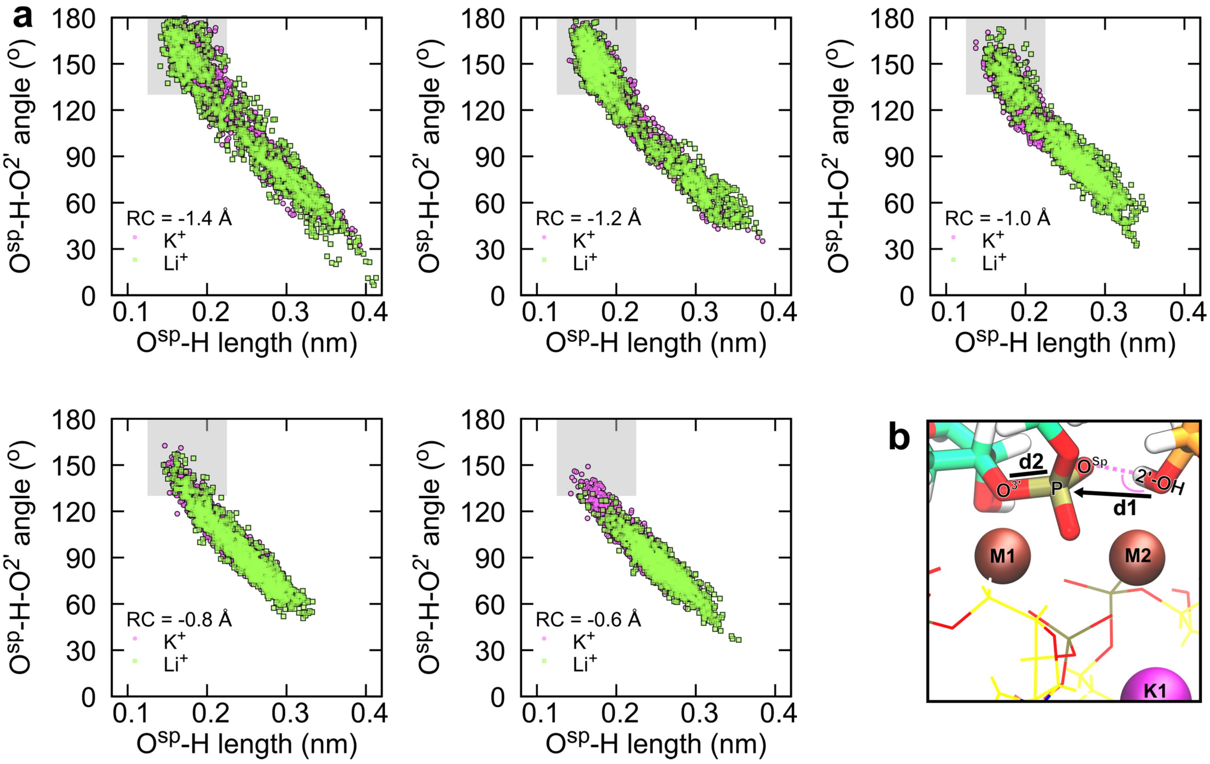

Supplementary Figure 15. a, The angle defined by *pro*-S_p O of the scissile phosphate group and the nucleophilic 2'-OH from BPA as a function of the distance between *pro*-S_p O and 2'-OH proton for different values of the reaction coordinate (RC). The area of values that allow for hydrogen bond formation is marked in grey. Violet dots correspond to simulations performed with the K^+ ion, while green dots show the distributions obtained when, instead, Li^+ was bound in the active site. **b**, Close up of the active site. The analysed distance and angle are marked in violet. The reaction coordinate is the difference between the breaking and forming bond (RC = d2 – d1). Source data are provided as a Source Data file.

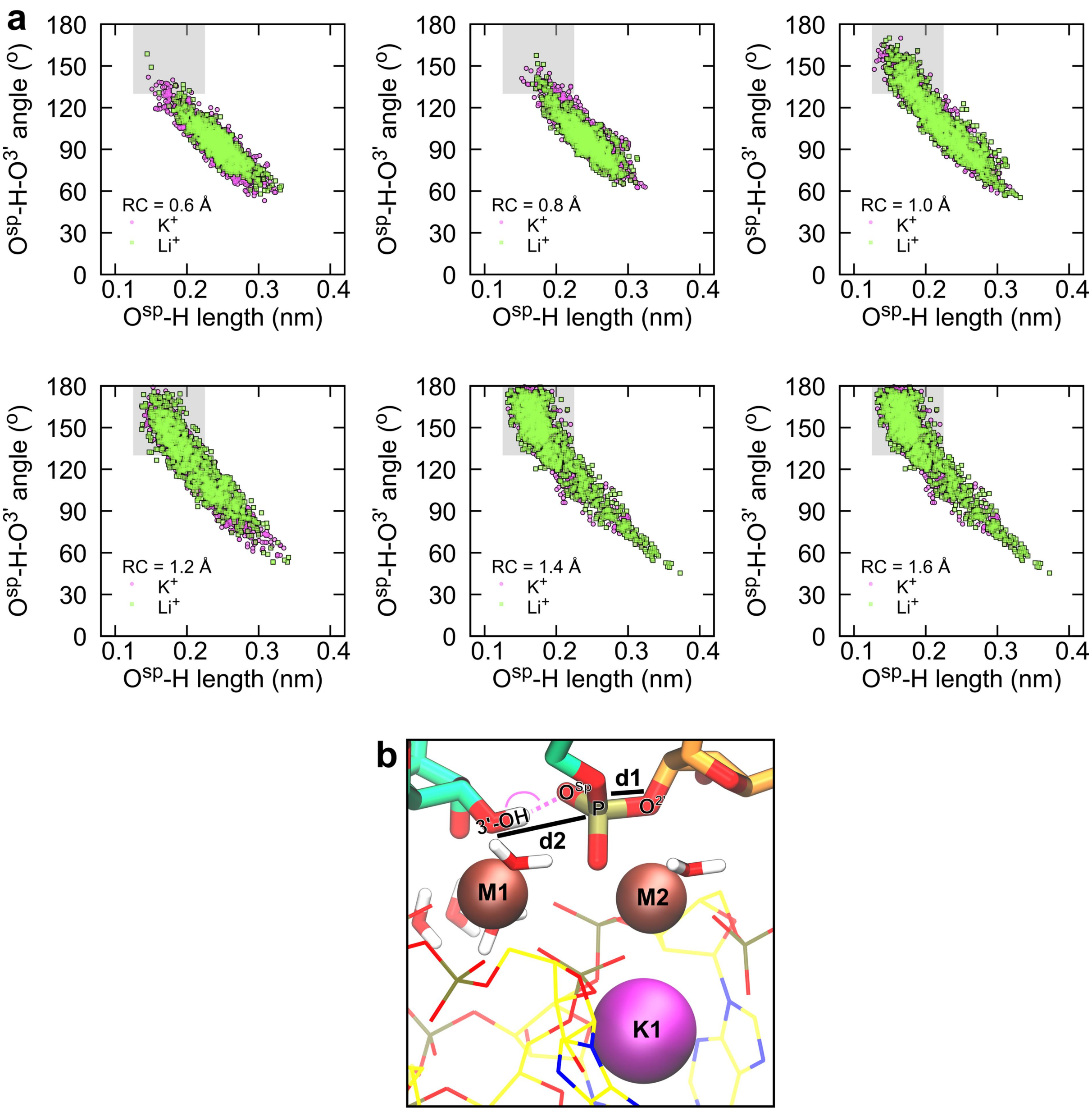

Supplementary Figure 16. a, The angle defined by *pro*-S_p O of the scissile phosphate group and the leaving hydroxyl group (3'-OH) in the branching reaction as a function of *pro*-S_p O and 3'-OH proton distance for different values of the reaction coordinate. The area of values that allow for hydrogen bond formation is marked in grey. Violet dots correspond to simulations performed with the K^+ ion, while green dots show the distributions obtained when, instead, Li^+ was bound in the active site. **b**, Close up of the active site. The analysed distance and angle are marked in violet. Reaction coordinate is the difference between the breaking and forming bond (RC = d2 – d1). Source data are provided as a Source Data file.

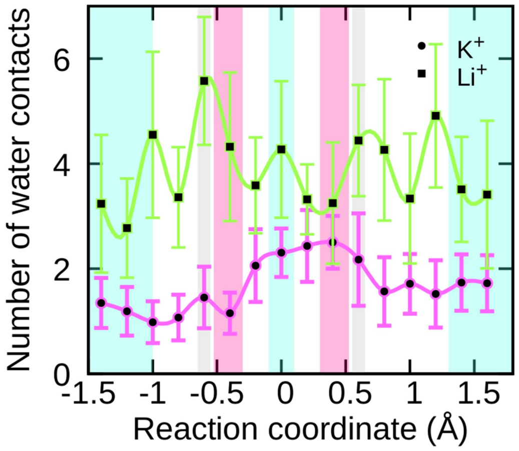

Supplementary Figure 17. The average number of water molecules in the vicinity of the monovalent ion as a function of the reaction coordinate. Cut off of 4 Å was used for the calculation. The results of simulations with K^+ are shown in violet, while the green line corresponds to simulations performed with the Li^+ ion. Data are presented as mean values +/- SD (n = 1000 frames). Source data are provided as a Source Data file

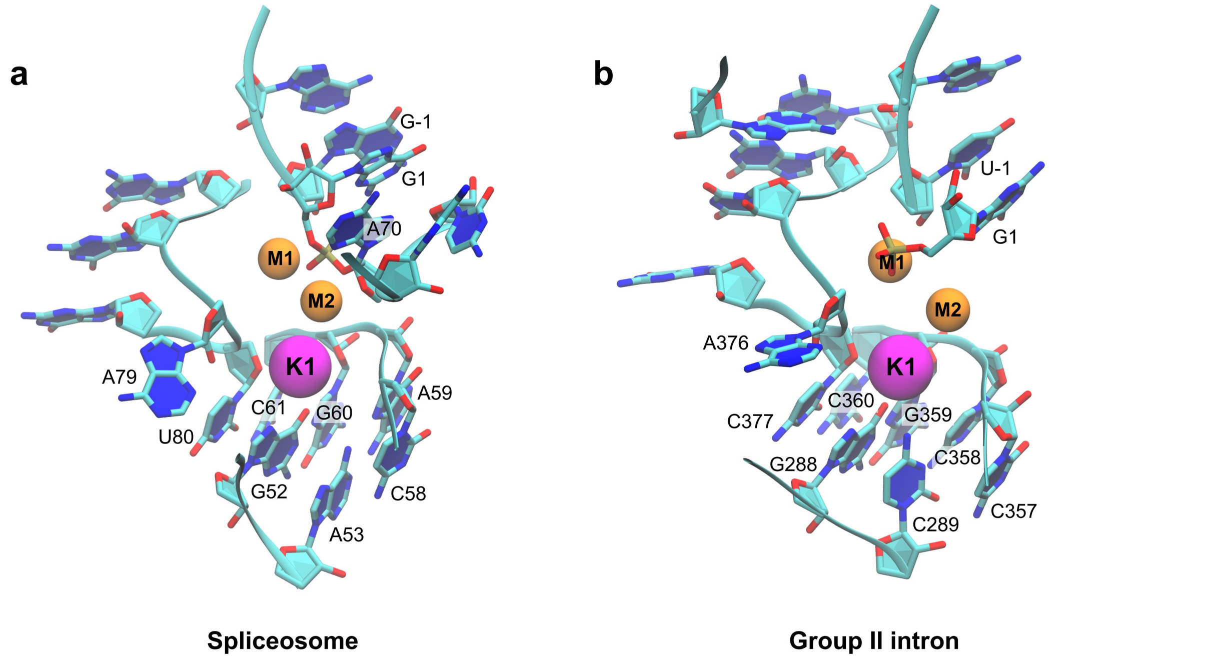

Supplementary Figure 18. Comparison of the active site in the (**a**) spliceosome from *Saccharomyces cerevisiae* (PDBID: 7B9V) and (**b**) group II intron from *Oceanobacillus iheyensis* (PDBID: 4FAR) right after the branching reaction reveals the K1 site is nearly identical, suggesting a common role of the potassium ion in the reaction mechanism.

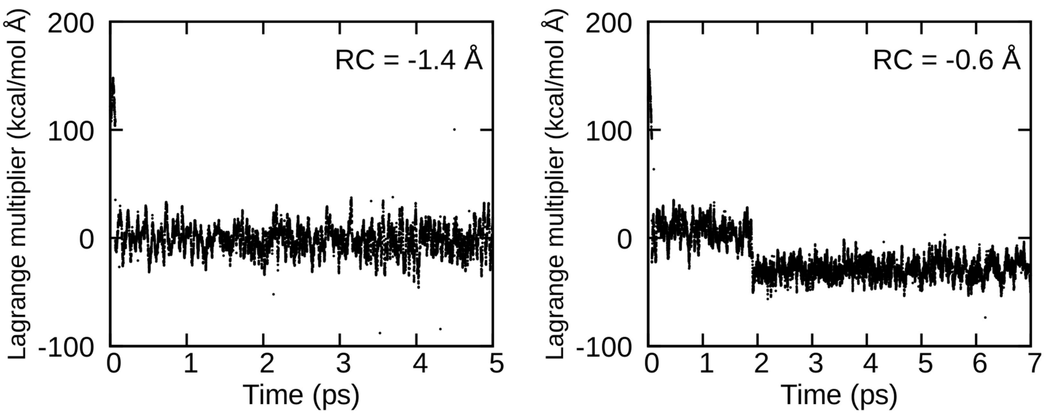

Supplementary Figure 19. Representative time evolutions of the Lagrange multiplier during biased QM/MM MD. At RC = -1.4 Å, the Lagrange multiplier was well-converged after 2 ps of simulation time. Instead at RC = -0.6 Å, where the first proton transfer is observed, the Lagrange multiplier needed longer time to converge. Therefore, to obtain reliable statistics the QM/MM MD simulations were extended to 7 ps and the mean force was calculated from the last 3 ps of the trajectory.

Supplementary Table 1. Composition of all simulated systems.

	**Total number of atoms**	**Total number of water molecules**	**Total number of neutralizing Na^+ ions**	**Simulation box size (nm^3)**
+Yju2, +Cwc25	550,487	165,480	191	16.7 × 20.3 × 16.2
+Yju2, -Cwc25 (K^+)	535,391	160,712	196	17.7 × 18.0 × 16.8
+Yju2, -Cwc25 (Li^+)	535,391	160,712	196	17.7 × 18.0 × 16.8
-Yju2, -Cwc25	533,509	160,716	206	17.7 × 18.0 × 16.7
