# Supplementary material for: Monovalent metal ion binding promotes the first transesterification reaction in the spliceosome
Source: Nat Commun. 2023 Dec 20;14:8482. doi: 10.1038/s41467-023-44174-2 (PMC10733407; doi:10.1038/s41467-023-44174-2)
Supplement: Supplementary file 3 — Description of Additional Supplementary Files [file 41467_2023_44174_MOESM3_ESM.pdf]

Description of Additional Supplementary Files:

Supplementary Data 1: XLS file containing a list of protonation states of ionisable residues. Chain identifiers and residue numbering are as in PDBID: 7B9V.

Supplementary Movie 1: The mechanism of the branching step of the splicing reaction as observed in hybrid quantum mechanical/classical molecular dynamics simulations.
