# Supplementary material for: Monovalent metal ion binding promotes the first transesterification reaction in the spliceosome
Source: Nat Commun. 2023 Dec 20;14:8482. doi: 10.1038/s41467-023-44174-2 (PMC10733407; doi:10.1038/s41467-023-44174-2)
Supplement: Supplementary file 6 — Reporting Summary [file 41467_2023_44174_MOESM6_ESM.pdf]

nature portfolio

Corresponding author(s): Alessandra Magistrato

Last updated by author(s): Nov 9, 2023

Reporting Summary

Nature Portfolio wishes to improve the reproducibility of the work that we publish. This form provides structure for consistency and transparency in reporting. For further information on Nature Portfolio policies, see our Editorial Policies and the Editorial Policy Checklist.

Statistics

For all statistical analyses, confirm that the following items are present in the figure legend, table legend, main text, or Methods section.

n/a	Confirmed	
☐	☒	The exact sample size (*n*) for each experimental group/condition, given as a discrete number and unit of measurement
☒	☐	A statement on whether measurements were taken from distinct samples or whether the same sample was measured repeatedly
☒	☐	The statistical test(s) used AND whether they are one- or two-sided<br>*Only common tests should be described solely by name; describe more complex techniques in the Methods section.*
☒	☐	A description of all covariates tested
☒	☐	A description of any assumptions or corrections, such as tests of normality and adjustment for multiple comparisons
☐	☒	A full description of the statistical parameters including central tendency (e.g. means) or other basic estimates (e.g. regression coefficient) AND variation (e.g. standard deviation) or associated estimates of uncertainty (e.g. confidence intervals)
☒	☐	For null hypothesis testing, the test statistic (e.g. *F*, *t*, *r*) with confidence intervals, effect sizes, degrees of freedom and *P* value noted<br>*Give P values as exact values whenever suitable.*
☒	☐	For Bayesian analysis, information on the choice of priors and Markov chain Monte Carlo settings
☒	☐	For hierarchical and complex designs, identification of the appropriate level for tests and full reporting of outcomes
☒	☐	Estimates of effect sizes (e.g. Cohen's *d*, Pearson's *r*), indicating how they were calculated

Our web collection on statistics for biologists contains articles on many of the points above.

Software and code

Policy information about availability of computer code

Data collection	System set up was accomplished with AmberTools20, H++ web tool (version 3.0) and the SOLVATE program (version 1.0). Classical molecular dynamics (MD) were performed with gromacs (version 2020.3). QM/MM MD simulations were performed with cp2k (version 9.1). Initial and final configurations of simulated systems have been deposited in Zenodo [https://doi.org/10.5281/zenodo.10089929].
Data analysis	Data were analysed with cpptraj part of AmberTools20, APBS software (version 1.5) and Python (version 2.7.18) packages MDtraj (version 1.9.3) and MDAnalysis (version 0.20.1). Figures were prepared with VMD (version 1.9.3), gnuplot (version 5.2), gimp (version 2.10.30) and inkscape (version 1.2). Cp2k inputs for running QM/MM MD simulations and custom python scripts employed for the analysis of collected molecular dynamics trajectories have been deposited in Zenodo [https://doi.org/10.5281/zenodo.10089929].

For manuscripts utilizing custom algorithms or software that are central to the research but not yet described in published literature, software must be made available to editors and reviewers. We strongly encourage code deposition in a community repository (e.g. GitHub). See the Nature Portfolio guidelines for submitting code & software for further information.

Data

Policy information about availability of data

All manuscripts must include a data availability statement. This statement should provide the following information, where applicable:

- Accession codes, unique identifiers, or web links for publicly available datasets
- A description of any restrictions on data availability
- For clinical datasets or third party data, please ensure that the statement adheres to our policy

All data in support of the findings of this study are available within the article and in the Supplementary Information. Source data are provided with this paper. Initial and final configurations of simulated systems have been deposited in Zenodo [https://doi.org/10.5281/zenodo.10089929]. Due to their large size, MD simulation trajectories are available from the corresponding author upon request. The experimental structures of the spliceosome from Saccharomyces cerevisiae in the C complex state and group II intron from Oceanobacillus iheyensis are available in Protein Data Bank under accession codes 7B9V [https://doi.org/10.2210/pdb7B9V/pdb] and 4FAR [https://doi.org/10.2210/pdb4FAR/pdb], respectively.

Research involving human participants, their data, or biological material

Policy information about studies with human participants or human data. See also policy information about sex, gender (identity/presentation), and sexual orientation and race, ethnicity and racism.

Reporting on sex and gender	Our research does not involve human participants.
Reporting on race, ethnicity, or other socially relevant groupings	Our research does not involve human participants.
Population characteristics	Our research does not involve human participants.
Recruitment	Our research does not involve human participants.
Ethics oversight	Our research does not involve human participants.

Note that full information on the approval of the study protocol must also be provided in the manuscript.

Field-specific reporting

Please select the one below that is the best fit for your research. If you are not sure, read the appropriate sections before making your selection.

[x] Life sciences [ ] Behavioural & social sciences [ ] Ecological, evolutionary & environmental sciences

For a reference copy of the document with all sections, see nature.com/documents/nr-reporting-summary-flat.pdf

Life sciences study design

All studies must disclose on these points even when the disclosure is negative.

Sample size	Classical MD simulations were performed for 100 ns, to relax the system before performing more accurate QM/MM MD simulations. Unbiased QM/MM MD simulations were performed for 5 ps for each system (after equilibration), as this is an appropriate time scale to assess the stability of metal coordination spheres. Time evolution of interatomic distances confirmed the active site was indeed well equilibrated. Average values and standard deviations were calculated over 1000 frames. Biased QM/MM MD simulations were performed for 5-7 ps, for each value of the reaction coordinate totaling in approximately 80 ps of simulation time. Analysis of Lagrange multipliers, needed for obtaining reaction free energy profiles, showed they were sufficiently converged after 2-4 ps. Average values and standard deviations of the Lagrange multiplier were calculated over the last 3 ps (6000 frames).
Data exclusions	No data were excluded from analysis.
Replication	While no formal replication was carried out due to computational costs, unbiased QM/MM MD simulations were performed with different combinations of step one specific splicing factors. No major effects on the active site geometry were observed, indicating simulations are reproducible.
Randomization	No randomization was performed. It is not relevant to molecular dynamics simulations performed here, since different reaction coordinate (RC) values are assigned to the same system.
Blinding	No blinding was performed as it cannot be implemented in our case, since the identity of each system (RC value) must be known to calculate free energy profiles via thermodynamic integration.

Reporting for specific materials, systems and methods

We require information from authors about some types of materials, experimental systems and methods used in many studies. Here, indicate whether each material, system or method listed is relevant to your study. If you are not sure if a list item applies to your research, read the appropriate section before selecting a response.

Materials & experimental systems

n/a	Involved in the study
☒	☐ Antibodies
☒	☐ Eukaryotic cell lines
☒	☐ Palaeontology and archaeology
☒	☐ Animals and other organisms
☒	☐ Clinical data
☒	☐ Dual use research of concern
☒	☐ Plants

Methods

n/a	Involved in the study
☒	☐ ChIP-seq
☒	☐ Flow cytometry
☒	☐ MRI-based neuroimaging
